# Assessing the cost-effectiveness of integrated case management of Neglected Tropical Diseases in Liberia

**DOI:** 10.1186/s12913-023-09685-0

**Published:** 2023-06-29

**Authors:** Tiawanlyn G. Godwin-Akpan, Karin Diaconu, Melissa Edmiston, John Solunta Smith, Fred Sosu, Stefanie Weiland, Karsor K. Kollie

**Affiliations:** 1 American Leprosy Missions, Greenville, SC USA; 2 Institute for Global Health and Development, Queen Margaret University, Edinburgh, Musselburgh, EH21 6UU Scotland; 3 Capitol Bye-Pass, University of Liberia – Atlantic Centre for Research and Evaluation, University of Liberia, Monrovia, Liberia; 4 Neglected Tropical Diseases Program, Ministry of Health, Oldest Congo Town, Monrovia, Liberia

**Keywords:** NTDs, Integrated approach, Case management, Cost-savings

## Abstract

**Background:**

In 2017, Liberia became one of the first countries in the African region to develop and implement a national strategy for integrated case management of Neglected Tropical Diseases (CM-NTDs), specifically Buruli ulcer, leprosy, lymphatic filariasis morbidities, and yaws. Implementing this plan moves the NTD program from many countries' fragmented (vertical) disease management. This study explores to what extent an integrated approach offers a cost-effective investment for national health systems.

**Methods:**

This study is a mixed-method economic evaluation that explores the cost-effectiveness of the integrated CM-NTDs approach compared to the fragmented (vertical) disease management. Primary data were collected from two integrated intervention counties and two non-intervention counties to determine the relative cost-effectiveness of the integrated program model vs. fragmented (vertical) care. Data was sourced from the NTDs program annual budgets and financial reports for integrated CM-NTDs and Mass Drug Administration (MDA) to determine cost drivers and effectiveness.

**Results:**

The total cost incurred by the integrated CM-NTD approach from 2017 to 2019 was US$ 789,856.30, with the highest percentage of costs for program staffing and motivation (41.8%), followed by operating costs (24.8%). In the two counties implementing fragmented (vertical) disease management, approximately US$ 325,000 was spent on the diagnosis of 84 persons and the treatment of twenty-four persons suffering from NTDs. While 2.5 times as much was spent in integrated counties, 9–10 times more patients were diagnosed and treated.

**Conclusions:**

The cost of a patient being diagnosed under the fragmented (vertical) implementation is five times higher than integrated CM-NTDs, and providing treatment is ten times as costly. Findings indicate that the integrated CM-NTDs strategy has achieved its primary objective of improved access to NTD services. The success of implementing an integrated CM-NTDs approach in Liberia, presented in this paper, demonstrates that NTD integration is a cost-minimizing solution.

**Supplementary Information:**

The online version contains supplementary material available at 10.1186/s12913-023-09685-0.

## Background

NTDs are a group of 20 or more diverse diseases disproportionately affecting poor and marginalized populations and rural communities. Health policymakers and funders relatively ignore NTDs compared to other public health issues [1]. Given that NTD interventions often receive limited resources, many persons affected have limited access to needed healthcare services. However, early diagnosis and management can significantly reduce the risk of associated morbidity and disability [2].

The 2012 World Health Organization (WHO) NTD Roadmap and the London Declaration on NTDs successfully drew attention and funding to NTDs as one of the targeted health priorities at national, regional, and global levels [3]. As a result, the support for NTDs has expanded; however, the priority is still disproportionately placed on preventive chemotherapy and transmission control (PCT) of NTDs, with most funding allocated to mass drug administration (MDA) rather than the case management of NTDs (CM-NTDs) [4]. This is due to the long treatment duration and routine activities needed to support CM-NTDs, including supervision, case detection, referral, diagnosis, reporting, follow-up care, and disability prevention. As a result, many countries still operate disease-specific or fragmented (vertical) programs focused on prevention, with limited resources allocated to reach persons affected by NTDs. The new 2021–2030 WHO Roadmap for NTDs prioritizes the integration of NTDs to improve access to management services for better treatment outcomes [1].

Liberia is one of the few countries that has implemented a comprehensive, integrated approach to the case management of NTDs. In 2017, the national NTD Program launched the first "National Strategic Plan for the Integration of Case Management of NTDs in Liberia (2016–2020)” [5]. The fifteen counties in Liberia are all co-endemic for Buruli ulcer (BU), clinical manifestations of lymphatic filariasis (hydrocele and lymphedema) and leprosy, as well as more recently yaws, which has re-emerged after more than twenty years, and the prevalence is still unknown [5]. Implementing the integrated strategy resulted in structural integration as leprosy was removed from the National Tuberculosis and Leprosy Control Program (NTBLP) to the national NTDs program. This integration was implemented in five of Liberia’s fifteen counties over three years, while the remaining ten counties continued fragmented (vertical) disease management. The implementation aimed to ascertain whether the integrated approach could facilitate improved access to care and universal health coverage for people with NTDs in Liberia. The WHO defines integration as “the organization and management of health services so that people get the care they need, when they need it, in ways that are user friendly, achieve the desired results and provide value for money” [6]. The fragmented (vertical) approach to case management of NTDs can be characterized by vertical disease programs without collaboration or coordination or sporadic intervention activities that are not integrated into routine services. This paper uses the latter definition when describing Liberia's fragmented (vertical) disease management.

Many scholars emphasize the need for integration at national and international levels to improve the effectiveness and efficiency of NTD healthcare delivery, such as Mitjà et al., primarily because the significant burden of NTDs exists in areas where health systems are fragile and under-resourced [7]. However, policymakers and global funders are still unclear about the benefits of an integrated approach compared to vertical disease management. While the benefits of integration (e.g., the potential for increased access to timely diagnosis of cases from the communities) have been widely discussed, there is a need to understand better how such integrated strategies can be implemented effectively and efficiently [7–9]. This research aimed to explore the cost-effectiveness of integrated NTD case management compared to fragmented (vertical) disease management, drawing on the experience of the integrated care project implementation in Liberia.

## Methods

### Design and research question

This study is a mixed-method economic evaluation that explores the cost-effectiveness of the integrated CM-NTDs approach implemented in Liberia compared to fragmented (vertical) disease management. (see description of models of care below).

The following research questions guided the evaluation:

1. What costs are associated with integrated CM-NTD implementation, and what are the main cost drivers?
2. How do the integrated CM-NTDs and fragmented (vertical) disease management compare in relation to their costs and effectiveness?

Methods used for this economic evaluation follow the guidance in the International Reference Case on Economic Evaluation [10].

### Models of care

#### Fragmented care: fragmented (vertical) disease management

**Fig. 1:**
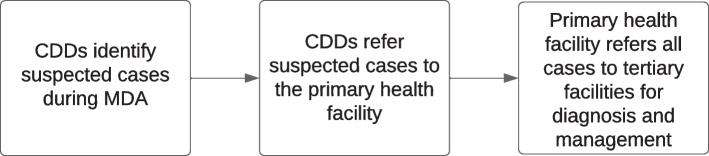
Overview of fragmented (vertical) disease management

Fragmented (vertical) disease management implies that active case search for NTDs is only conducted in mass drug administration (MDA) campaign periods. This means a case search is conducted twice yearly for 2–3 weeks. When MDA ends, the health facilities rely on patients self-reporting to the health facility. Healthcare workers, community health assistants (CHA), and volunteers (CHV) receive training on NTDs during this period; training focuses on the disease for which MDA is being conducted without providing information on the diagnosis and management of NTDs requiring case management at the primary healthcare level. Therefore, under this approach, care for NTDs that need case management is ad-hoc and not routine due to limited knowledge among primary healthcare workers and the unavailability of NTD drugs and commodities. As a result, affected persons must travel far distances to tertiary health facilities for diagnosis and management, as seen in Fig. 1. At this point, depending on the training of clinicians encountered, diagnosis and treatment may be initiated.

#### Comparator intervention: integrated case management

**Fig. 2:**
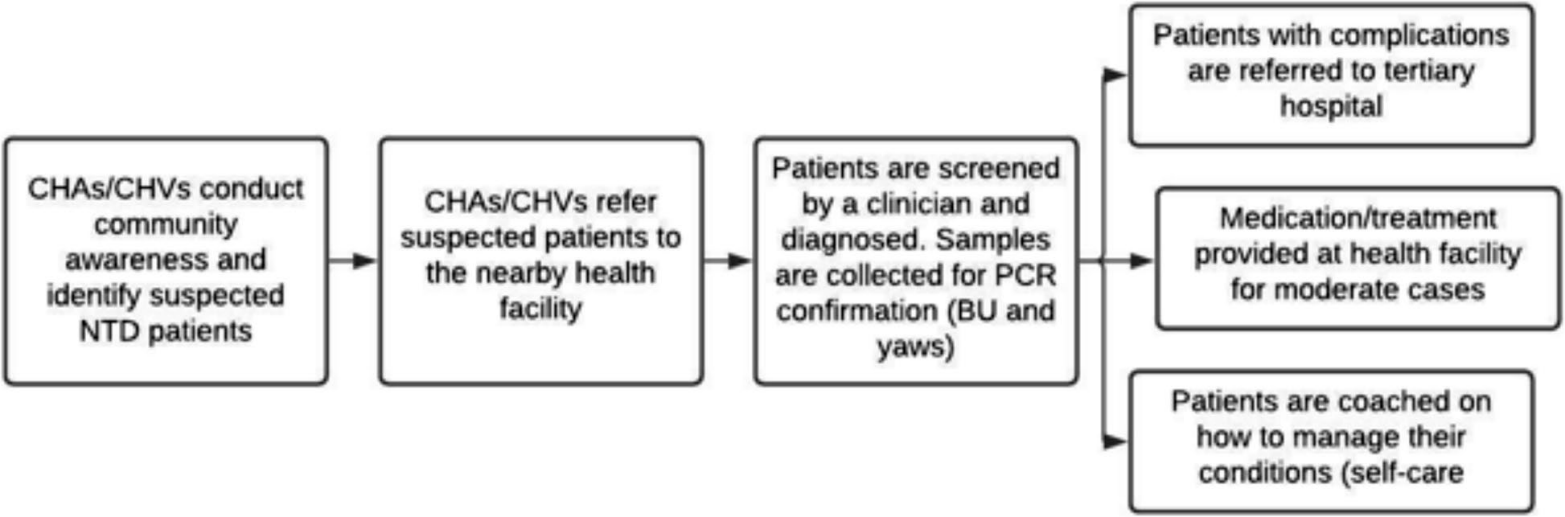
Overview of integrated case management intervention

The integrated case management referral pathway involves activities shown below in Fig. 2. It starts with CHAs/CHVs conducting daily door-to-door visits and community awareness to identify people showing signs and symptoms of NTDs and refer them to the nearest health facilities. These CHAs/CHVs provide health education to promote awareness-raising and stigma reduction relating to NTDs in their communities and promote good hygiene practices to reduce the risk of disability from NTDs or other conditions. If a person is then assumed to have an NTD, CHA/CHVs refer the case to the nearest health facility, where cases are then clinically diagnosed by trained clinicians and placed on treatment. Clinicians also conduct health education for home-based self-care. For those requiring testing (BU and yaws), samples are collected and tested for polymerase chain reaction (PCR) confirmation. Patients with complications are referred to county hospitals or tertiary health facilities for specialized care. Counter-referrals to primary healthcare facilities occur, including disbursal of home-based care kits for conditions. When persons are diagnosed to have an NTD at the health facility, the CHA/CHV receives a case-finding incentive. The CHA/CHV also continues to visit NTD patients in the community while on treatment. While these CHAs/CHVs' daily tasks are not limited to the NTDs program as they serve the entire health system in various capacities, including distributing mosquito nets, case identification for other conditions, and health education in the community integrating NTDs into the existing health system structure at all levels also make NTDs a priority. Since NTDs are now reported through the Health Information System (HIS), it is a priority for all health workers.

### Data collection

To understand the cost drivers of the integrated CM-NTD model of care, cost data was sourced from the national CM-NTD annual budget and financial reports. These data capture expenditures across all five integrated CM-NTDs intervention counties and multiple health system levels – i.e., spending at the community level, direct facility level, and above facility-level costs (such as those incurred in program management and oversight) are considered. The fragmented (vertical) disease management cost was sourced from annual MDA budgets and financial reports.

Primary data were collected to determine the relative cost-effectiveness of the integrated program model vs. fragmented (vertical) care. The research team traveled to four counties, two (2) intervention counties (Bomi and Bong) and two non-intervention counties (Margibi and Grand Cape Mount). These counties were purposively selected because they are all co-endemic for CM-NTDs and share similar geographic and environmental characteristics. Due to financial constraints, the five intervention counties could not be visited.

Researchers thus visited two (2) districts in each of the two selected intervention counties (Bong and Bomi) and one (1) district in each of the selected intervention counties (Grand Cape Mount and Margibi) for data collection. The team of researchers included three (3) research assistants, one (1) data analyst, and one (1) supervisor. The researchers engaged the fieldwork simultaneously in undertaking key informant interviews (KIIs) at the district and county levels while also reviewing and extracting relevant data from county-level program documents. A total of 32 interviews were conducted in the study counties: six (6) at the national level, thirteen (13) in the two intervention counties, and thirteen (13) in the non-intervention counties. For each KII, a topic guide was developed as a data collection tool to tailor the questions to the roles of each group. For example, the CHA/CHVs topic guide considered the referral pathway when a case is found, the basic care and coaching services provided, and the follow-up mechanism. They were also asked about the training they received as CHAs/CHVs, the duration of the training, the impact, and the relevance of the training with respect to their jobs. The data collectors used Dictaphones to record the KIIs and notepads for notetaking: an interviewer and a notetaker conducted each interview.

Effectiveness data were sourced from annual technical reports of the program over the 2017–19 period, covering two intervention counties (Bong and Bomi) and two non-intervention counties (Margibi and Grand Cape Mount). Cost data was sourced from the integrated CM-NTDs expenditure data used in Component 1 above, and cost data was sourced from Ministry of Health records across Grand Cape Mount and Margibi counties. Data on the population of Liberia and the incidence of NTDs is sourced from global literature (see references below).

### Data analysis

The consultants transcribed all KIIs and coded available data. Descriptive codes focused on issues of NTD awareness raising, NTD service processes, care pathways, roles and duties of staff involved in NTD service delivery at diverse health system levels (ranging from community to facility, district, and county to national level), and patient experiences. As interviews were purposively conducted across diverse counties, the focus was on comparing NTD services between implementation vs. non-intervention counties using the constant comparison method. Review of descriptive codes, annotation of transcriptions upon repeat reading, and assessment of materials allowing for abstract broader themes relating to Integrated CM-NTDs implementation desired theory of change as understood by implementation county participants; NTD service delivery status quo in non-intervention counties; barriers and facilitators to integrated CM-NTDs implementation.

All cost data collected was first recategorized based on the purpose for which expenditures were incurred and the type of expenditure and level at which expenditure-related activities were due to take place (see Table 1 for definitions). Each cost incurred in the integrated CM-NTDs or fragmented (vertical) disease management counties was first assigned activity and further assigned a cost type.

**Table 1:** Description of cost categories

Domain	Categories	Definition
Activity type for which costs were incurred	Data Management	Any costs associated with reporting, collation, and/or curation of data
	Operating Costs	Refers to direct implementation of interventions and includes costs relevant to patient-focused activities (e.g. surveillance and awareness-raising and costs related to that such as transportation, also materials such as home-based care kits or stationery)
	National Program Oversight of the implementation of activities	Expenditure associated with high-level program oversight – e.g. national-level monitoring and evaluation activities
	Program Staffing & Motivation	Refers to salary costs and incentives paid out to any of the staff involved in NTD service delivery
	Supervision & Monitoring in the counties	Relates to expenditures incurred for conducting routine program supervisory and monitoring activities
	Training & Capacity building	Expenditure relating to all training activities – including peer exchanges and related costs such as travel, per diems, and/or meeting costs
Type of cost	Patient Direct (as incurred by the health system)	Includes costs for home-based care kits, laboratory supplies used for patients, medications, and other materials directly used in patient care
	Materials	All expenditures broadly related to material items used for NTD service delivery (not directly used for patients), including communication, maintenance costs for vehicles, stationery, transportation materials (e.g. vehicle tires)
	Allowances	Includes per diems and transportation allowances
	Reporting	Communication costs for submitting reports
	Meetings	Space rental and refreshment costs
	Evaluation	Costs of any surveys
	Staff costs	Staff salaries and incentives
	Bank charges and overheads	Charges and overheads as incurred due to transfers
The level at which activities incurring costs take place	Community	Within communities
	Facility	Within the health facility
	District	At district levels – e.g. District Surveillance Officers
	County	At the county level – e.g. county-level reporting
	National	At the national level – e.g. overarching program meetings
	Mix	A mix of levels as indicated above

All expenditure was directly incurred in US$, so no exchange rate adjustments were made. Once costs were categorized, the annual total cost of the program was calculated. Drawing on these financial costs, a descriptive analysis of cost drivers for the integrated CM-NTDs implementation was conducted.

### Analysis of the economic evaluation

The evaluation assumes a health system perspective: i.e., only costs directly incurred by the health system as available in the costing documentation reviewed are captured. This evaluation captures no indirect costs and no patient costs.

### Time horizon

To project the effects of care as usual and integrated CM-NTDs in the future, the evaluation is launched based on current values for the years 2021–25. A five-year time horizon was selected because this corresponds to a policymaker and donor-specific integrated CM-NTDs implementation period and is thus likely to offer sufficient information on medium-term expected benefits and costs of interventions [11].

### Discount rate

A discount rate of 5% was applied for both the calculation of cost and effects; while a discount rate of 3% is usual, Liberia is a low-income country and may benefit from a higher discounting rate [11]. Discounting is necessary as future costs and effects are valued less than immediate ones (as standard for economic evaluation). All costs and effects are first transformed to their present value to allow them to be aggregated.

### Estimating effectiveness

To assess the comparative effectiveness of the integrated CM-NTDs approach vs. fragmented (vertical) disease management, we considered the average number of NTD patients diagnosed divided by the total population per implementation year as the primary outcome. The result reflects the probability that an NTD-affected person has access to care and utilizes at least diagnostic services within the healthcare system within an average year. The relative probabilities for this outcome are estimated for integrated CM-NTDs from the implementation county (five counties) and fragmented (vertical) care from the non-implementation county (10 counties) from data available from 2017 to 2020.

We also present analyses that refer to the average number of NTD patients completing treatment (or on self-care in case of lymphoedema) divided by the total patients diagnosed per implementation year. This outcome reflects the probability that an NTD patient can receive and complete the requisite treatment (whether one-off or home-based ongoing care) for their condition within an average year. The relative probabilities for this outcome are estimated for integrated CM-NTD from the implementation county (two counties) and fragmented (vertical) care from the non-implementation county (two counties) data available over 2017–2019. However, readers should interpret these estimates cautiously as they derive the following analyses with extreme assumptions, including i) that the data of two counties is representative of the patient population; ii) that on average most patients complete treatment within a year (when some could feasibly in much longer time frames, up to two years).

A gross costing approach was used to calculate an average yearly cost per the primary outcomes. To capture the actual economic costs of the program, the present value of costs for all years was calculated using the discount rate identified above.

Additional file 1: Appendix 1 offers an overview of the decision-analytic model created to estimate the comparative costs and effectiveness of the integrated CM-NTDs vs. fragmented (vertical) care approach. The main estimates presented above are based on a decision-tree model capturing the relative probabilities of NTD-affected persons being seen and diagnosed by the health system and further completing treatment. The decision models illustrate the costs associated with each outcome (from the health system's perspective).

## Results

### The cost drivers of the integrated CM-NTD implementation

Table 2 provides an overview of the expenditure incurred (financial costs) by the integrated CM-NTDs implementation across 2017–2019. Figure 2 offers a graphic overview of the total spending for each program purpose shaded in grey (Table 2).

**Table 2:** Overview of costs incurred by CM-NTDs program (purpose and type of cost)

The purpose for which cost deployed and cost type	Year			Grand Total
	**2017**	**2018**	**2019**	
**Data Management**	**$0 (0%)**	**$2,704 (1%)**	**$4,796 (5%)**	**$7,500 (1%)**
Materials	$0 (0%)	$1,250 (0%)	$4,796 (5%)	$6,046 (1%)
Reporting	$0 (0%)	$1,454 (0%)	(0%)	$1,454 (0%)
**Operating costs**	**$100,544.7 (28%)**	**$80,269.44 (24%)**	**$15,469.44 (16%)**	**$196,283.58 (25%)**
Allowances	$2,810 (1%)	$3,214 (1%)	$2,356 (2%)	$8,380 (1%)
Bank charges and overheads	$2,978 (1%)	$3,550 (1%)	$6,163.44 (6%)	$12,691.44 (2%)
Materials	$6,8913 (19%)	$34,294.49 (10%)	$3,930 (4%)	$107,137.49 (14%)
Meetings	(0%)	$4,907.75 (1%)	(0%)	$4,907.75 (1%)
Patient direct	$25,843.7 (7%)	$34,303.2 (10%)	$3,020 (3%)	$63,166.9 (8%)
**National Program Oversight of the implementation of activities**	**$24,907.82 (7%)**	**$18,380.7 (6%)**	**$16,260 (16%)**	**$59,548.52 (8%)**
Allowances	$4,207.32 (1%)	$1,152 (0%)	(0%)	$5,359.32 (1%)
Evaluation	$7,900.5 (2%)	$10,489 (3%)	$16,260 (16%)	$34,649.5 (4%)
Materials	(0%)	$100 (0%)	(0%)	$100 (0%)
Meetings	$12,800 (4%)	$6639.7 (2%)	(0%)	$19,439.7 (2%)
**Program Staffing & Motivation**	**$143,800 (40%)**	**$154,140 (46%)**	**$32,700 (33%)**	**$330,640 (42%)**
Staff costs	$143,800 (40%)	$154,140 (46%)	$32,700 (33%)	$330,640 (42%)
**Supervision & Monitoring in the counties**	**$15,230.75 (4%)**	**$33,959.65 (10%)**	**$11,762.3 (12%)**	**$60,952.7 (8%)**
Allowances	$15,230.75 (4%)	$33,959.65 (10%)	$11,593.3 (12%)	$60,783.7 (8%)
Meetings	(0%)	(0%)	$169 (0%)	$169 (0%)
**Training & Capacity building**	**$73,713 (21%)**	**$42,987.5 (13%)**	**$18,231 (18%)**	**$134,931.5 (17%)**
Allowances	$67,760 (19%)	$27,816.5 (8%)	$18,231 (18%)	$113,807.5 (14%)
Meetings	$5,953 (2%)	$15,171 (5%)	(0%)	$21,124 (3%)
**Grand Total**	**$358,196.27 (100%)**	**$332,441.29 (100%)**	**$99,218.74 (100%)**	**$789,856.3 (100%)**

**Fig. 3:**
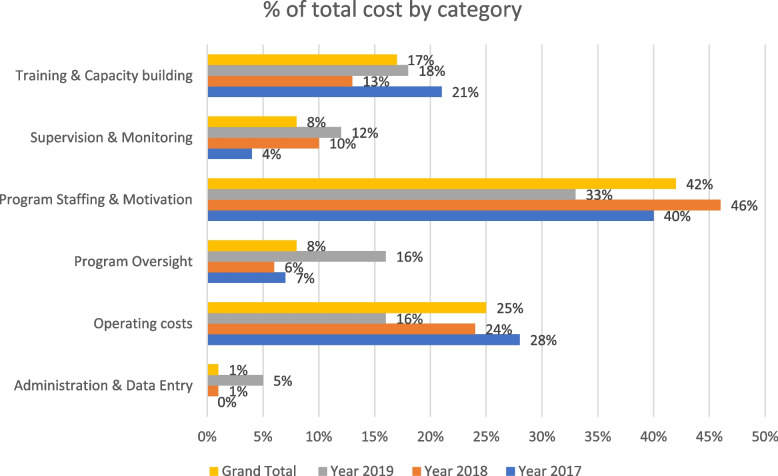
Overview of integrated CM-NTDs expenditure over 2017–19 by activity purpose

Table 2 and Fig. 3 suggest that the program’s expenditure primarily comprises staff costs (about 40%) allocated to salaries and incentives, decreasing over time. In 2017, high up-front training costs were recorded, but these go down over time. The reduction in cost after the first year of training is due to the timing of the initial training, which was conducted between 2017 and early 2018, with more health facilities covered in 2017. However, Key informant interviews (KII) suggest refresher training took place in 2019, mainly after the first round of training was conducted in 2017 and 2018. The refresher training covered the gaps for health facilities with new staff since the initial training and those health facilities that were identified to have challenges in diagnosing and managing NTDs cases through supervision. As a result, the cost of training in the subsequent years is lower than the initial cost at the start of the implementation. Operating costs (in this case, expenditure allocated specifically against medication, staff transport, and the program's materials (as necessary for sensitization) decrease over time. Notably, values drastically reduced operating costs. Surprisingly, with the assumption that integrated CM requires heavy oversight, the program involves relatively modest investment into oversight and monitoring, again suggesting relative efficiency of operations.

CHVs and CHAs in intervention counties report increased responsibilities and activities in relation to NTDs, focused explicitly on active case finding and activities to raise awareness of NTDs and reduce stigma.

*“When I am going out in the communities to do my awareness, I inform my boss and whatever I see I report to my boss and he will follow up to see and do what is necessary. (For awareness) I use flyers that have the picture of the diseases tell the people that this is the diseases I am talking so if you anyone who has it let me go and see. I do awareness one time a week and is mostly on Saturdays or sometime Sunday.” (Bomi County, Community health volunteer when asked about awareness raising)*

The ability of CHA and CHVs to carry out their role relies on the enhanced training and sensitization materials they received as part of the integrated CM-NTDs intervention. The training was provided at county levels, covering multiple days (usually 5 for health workers and 1–2 days for CHA/CHVs) and focused on the four endemic NTDs requiring case management in Liberia. Participants directly credit training with enhancing referrals to primary care facilities.

Health workers noted that the integrated CM-NTDs intervention also increased their and the facility’s capacity to manage NTDs due to several staff receiving training and materials assisting with case management.

*“I was trained on how to identify and treat those CM cases and there was also new ledger introduced on how to record patients’ information. So we have had about two to three trainings for last year to this year.” (Officer in Charge (OIC), Bong)*

The table and figure also suggest a gradual evolution in the program’s activities, indicative of relative efficiency. Qualitative data collection indicates that minimal expenditure is recorded against program administration and data entry. Alongside this evaluation, this is not surprising as the integrated CM-NTDs approach includes integrating NTDs data into the Health Information System (HIS), which was successfully done in 2019, thus reducing the burden of data collection and management on the program staff.

### How do integrated CM-NTDs and vertical disease management compare in terms of cost-effectiveness?

Based on the actual program data recorded, Table 3 summarizes the financial and economic costs and total effectiveness outcomes recorded across 2017–2019. Table 3 illustrates that in the two counties offering fragmented (vertical) disease management, approx. US$ 325,000 was spent on the diagnosis of 84 persons and treatment of twenty-four persons suffering from leprosy, Buruli ulcer, lymphoedema, and hydrocele. In contrast, while approx. 2.5 times as much was spent in implementation countries, and 9–10 times more patients were diagnosed and treated here.

**Table 3:** Total costs and effects of integrated CM-NTDs vs. fragmented (vertical) disease management

Costs and effects	Fragmented (vertical) Disease Management				Integrated case management			
	2017	2018	2019	Total	2017	2018	2019	Total
Total financial cost (US$)	$128,348	$54,098	$143,218	$325,664	$358,196	$332,441	$99,219	$789,856
Total economic cost (US$)	$122,236	$49,068	$123,717	$295,022	$341,139	$301,534	$85,709	$728,382
Total persons diagnosed (unadjusted^a^)	27	36	21	84	466	445	234	1145
Total persons diagnosed (adjusted^a^)	25	33	18	77	443	404	202	1050
Total persons on treatment (unadjusted^a^)	15	1	8	24	253	203	262	718
Total persons on treatment (adjusted^a^)	14	1	7	22	241	184	226	651

^a^Economic costs and adjusted figures consider discounting; financial and unadjusted figures do not account for this. Effects refer to persons diagnosed or on treatment; they are calculated as a total across patients with leprosy, Buruli ulcer, lymphoedema, and hydrocele (See Additional file 1: Appendix 1)

In non-intervention counties, CHAs and CHVs noted they had limited skills and knowledge of the signs for identifying the targeted NTDs. While some staff had received some NTD training, this was generally inconsistent. Community health services supervisors (CHSS) noted that most activities relating to NTDs were only taking place during the mass drug administration campaign periods. However, this is particularly challenging as not all NTDs can benefit from MDA. Further, facility staff noted they also need to gain more skills relating to NTD management. They said that, in most cases, they are simply responsible for referring NTD patients to higher levels of care.

*“When those cases are identify especially buruli ulcer we directly call the NTD Focal Person. Many times he send some dressing materials if we able to manage it at the facility level or at the community level. He send some dressing materials like gloves, bandage and pofidine. Sometimes he send those things, but it can actually be less for some of those cases we find in the community. Sometimes he can manage to send some materials, but at the National level those things are not enough, but at times base on the urgency of the case when we call for it he can send few to manage and sometimes when we are not able, we send the case or refer to a higher health facility. Sometimes we identify cases like hydrocele. During the time of the campaign I was able to identify three cases and we reported them, but there was some delay in the management. I made a follow up, educated the patients and at the end they went to Montserrado and did their surgery. But when it comes to buruli ulcer mostly it’s a major case in the various communities which we sometimes see. We can manage them sometimes if they send materials.” (Community Health Services Supervisor, Cape Mount)*

The qualitative data revealed that contrast to pilot counties, several other elements are also not present in the Fragmented (vertical) program:

- No incentives exist for community health volunteers, assistants, or health facility staff in relation to roles performed.
- No sensitization tools relating to the different types of NTDs are available.
- No NTD reporting forms or tools.

Table 4 summarizes the relative costs per patient diagnosed and treated under each program. The price of a patient being diagnosed under the fragmented (vertical) disease management intervention model is approx. five times higher than that under the integrated CM-NTDs model. Similarly, providing treatment under fragmented (vertical) disease management is approximately ten times as costly as the integrated CM-NTDs program.

**Table 4:** Costs and people for each program modality^a^

Costs/person for each program	Fragmented (vertical) care	CM-NTD
Unadjusted		
Cost (US$)/person diagnosed	$3,877	$690
Cost (US$)/person on treatment	$13,569	$1,100
Adjusted		
Cost (US$)/person diagnosed	$3,831	$694
Cost (US$)/person on treatment	$13.410	$1,119

^a^Adjustment refers to discounting as per the methods section

## Discussion

Our findings suggest that the main cost driver of the CM-NTD integrated care model relates to staffing and motivation (making up about 40% of expenditure) and operating costs (25% on average, mainly incurred for material expenses). Prices for the program have decreased from 2017 onward, suggesting increased efficiencies in implementing the program.

Further, our findings suggest that fragmented (vertical) disease management is less cost-effective than integrated care. Under fragmented (vertical) care, approx. US$ 3,831 is spent for diagnosis and $13,410 for the care of each NTD patient. In contrast, costs are five times lower for diagnosis under the integrated care approach and ten times lower for treatment under the latter approach.

The evaluation indicates that the integrated CM-NTDs strategy has achieved its primary objective of ensuring improved access to NTD services. This finding is supported by Marais and Petersen's study, where the integration of mental health into the integrated chronic disease management (ICDM) at the primary healthcare level was seen to improve access and reduce stigma [12]. The Integrated Case-Management intervention is overarchingly able to secure increased benefits for the population at a substantively lower cost. This is similar to findings on integrated MDA programs compared to stand-alone MDA in sub-Saharan Africa, where savings of 26–47% can be projected from such integration [13]. These findings show that integrated CM-NTD expansion results in more significant benefits in patients diagnosed and treated. Increased diagnosis rates and treatment due to integration found in this study are similar to a study conducted in Cote D’Ivoire [14].

We acknowledge several limitations to the study analysis and note that our findings should be interpreted cautiously, albeit optimistic for integrated care approaches. We proceeded pragmatically, using data available from routine sources. The evaluation focuses on all NTD patients affected by leprosy, lymphatic filariasis (and burden of lymphoedema and hydrocele due to this), and Buruli ulcer. While calculations were conducted to estimate the incidence of each of these NTDs, sub-group analyses for each of the specific NTDs could not be undertaken as the integrated CM-NTD implementation targets explicitly the integration of care delivery across these diseases, and as such, costs cannot be separated by disease category. The lack of cohort-based patient data on treatment poses a challenge for accurately calculating treatment completion rates. We presented estimates relating to this outcome given potential clinical interest but urge readers to consider this information highly uncertain and hope that interventions intended to strengthen routine information systems can help with conducting more detailed analyses in the future. Further, we could not identify the cost drivers of the fragmented care (fragmented (vertical)) disease management model due to missing data. This should be a priority for further research in Liberia.

Integrated interventions strengthen NTD service delivery, a conclusion supported by the qualitative study. While integrated CM-NTD approaches have specifically been implemented in counties with a high NTD burden in Liberia, this still suggests that a sizeable undetected burden of NTDs in non-intervention counties still needs to be addressed. This evaluation indicates that NTD integration is a cost-minimizing solution compared to fragmented (vertical) NTD case management. This study provides evidence for global policymakers to prioritize investment in integrated CM-NTDs as it is done for MDA.

## Supplementary Information

**Additional file 1. **Decision tree. 

**Additional file 2. **Analytic assumptions.

## Data Availability

All data generated or analyzed during this study are included in this published article [and its supplementary information files].

## Abbreviations

ALM: American Leprosy Missions
BU: Buruli Ulcer
CHA: Community Health Assistants
CHV: Community Health Volunteers
CM-NTDs: Case Management Neglected Tropical Diseases
FGD: Focus Group Discussion
HIS: Health Information System
ICDM: Integrated Chronic Disease Management
IRB: Institutional Review Board
KII: Key Informant Interviews
MDA: Mass Drug Administration
NTDs: Neglected Tropical Diseases
PCR: Polymerase chain reaction
PCT: Preventive chemotherapy
UL-PIRE: University of Liberia Pacific Institute for Research and Evaluation
WHO: World Health Organization
