# Supplementary material for: Assessing the cost-effectiveness of integrated case management of Neglected Tropical Diseases in Liberia
Source: BMC Health Serv Res. 2023 Jun 29;23:705. doi: 10.1186/s12913-023-09685-0 (PMC10308665; doi:10.1186/s12913-023-09685-0)
Supplement: Supplementary file 1 — Additional file 1. Decision tree. [file 12913_2023_9685_MOESM1_ESM.docx]

Persons with access


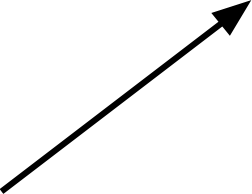
Integrated CM


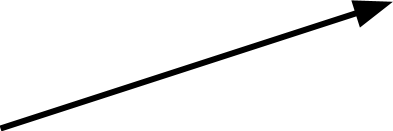

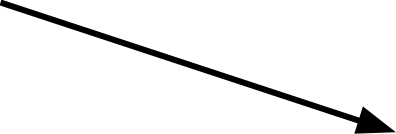


Persons without access

NTD conditions (incidence)

Persons with access


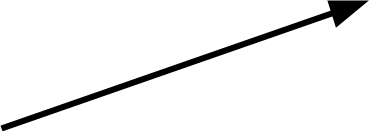

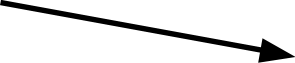


Vertical programme


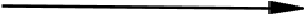

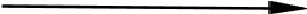
Persons without access


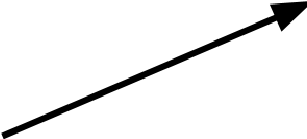

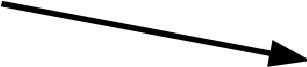


Disease specific trees - to understand logic


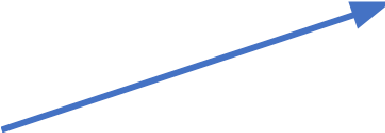


Diagnosed (confirmed)

Integrated CM


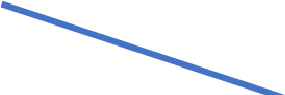

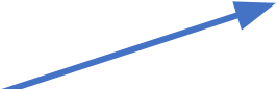


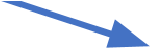
Un-diagnosed

Leprosy

Diagnosed (confirmed)

Vertical programme


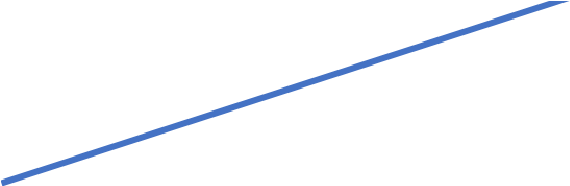

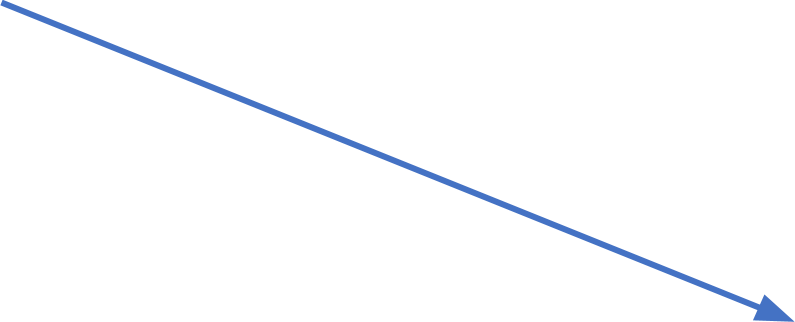

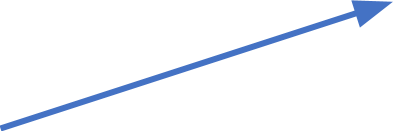

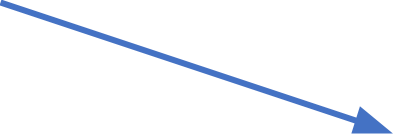


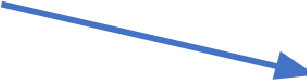

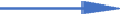

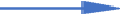

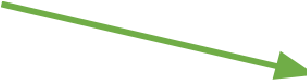

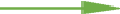
Un-diagnosed


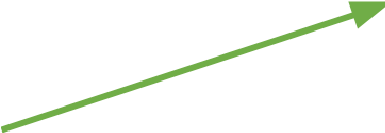


Diagnosed (clinically confirmed)

Integrated CM


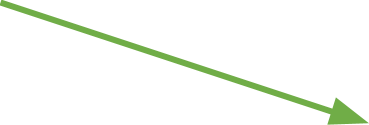
Un-diagnosed


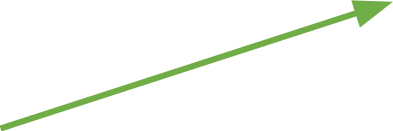

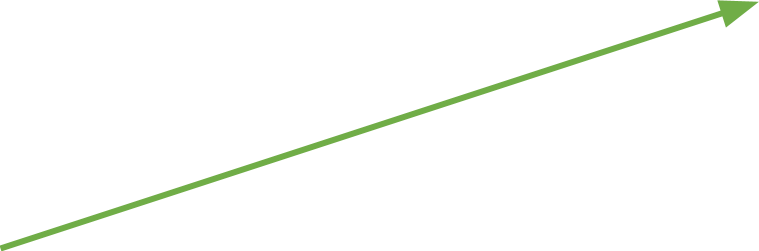

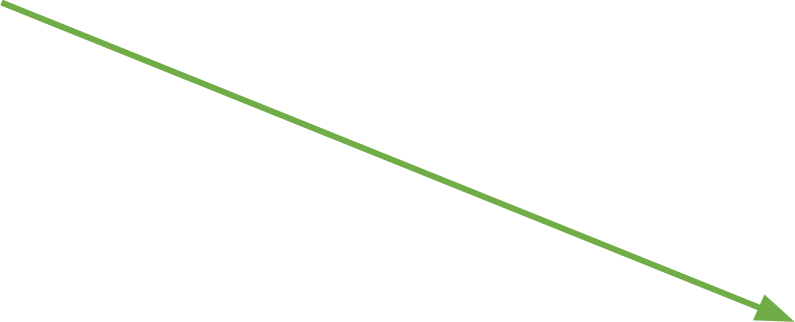


Buruli ulcer

Diagnosed (clinically confirmed)


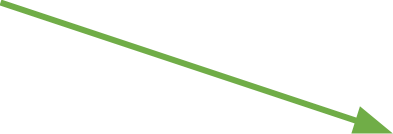
Vertical programme

Un-diagnosed


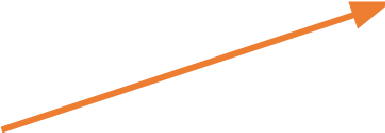


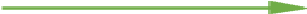

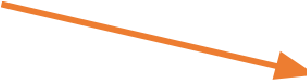

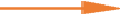

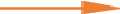
Diagnosed (confirmed)

Integrated CM


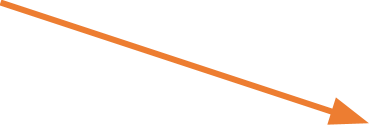
Un-diagnosed

Lymphoedema

Diagnosed (confirmed)

Vertical programme


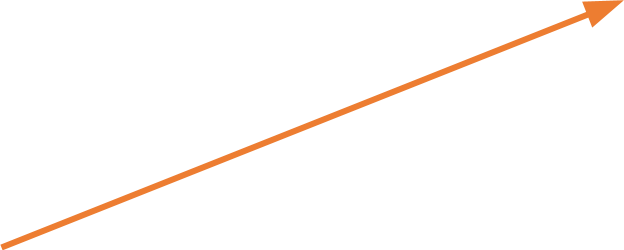

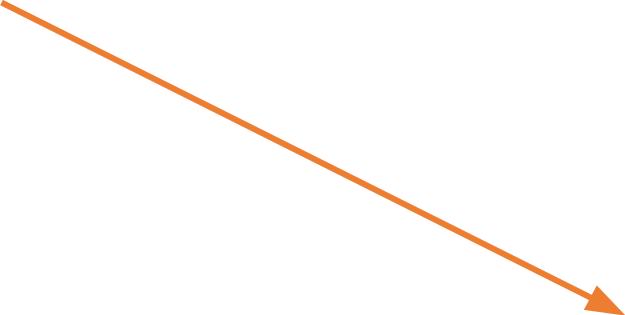

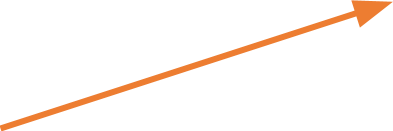

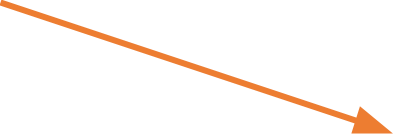


Un-diagnosed


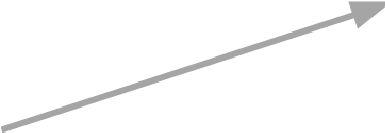


Diagnosed (confirmed)

Integrated CM


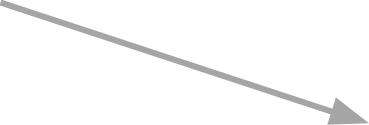
Un-diagnosed


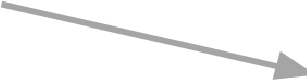

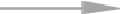

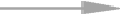
Hydrocele

Diagnosed (confirmed)

Vertical programme


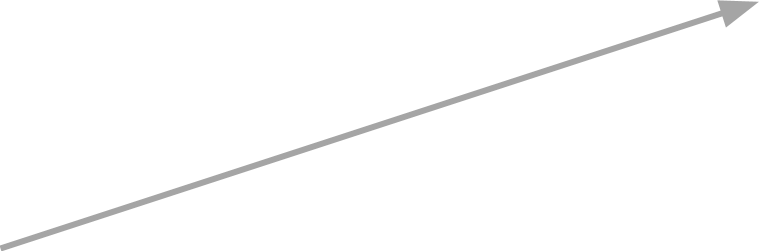

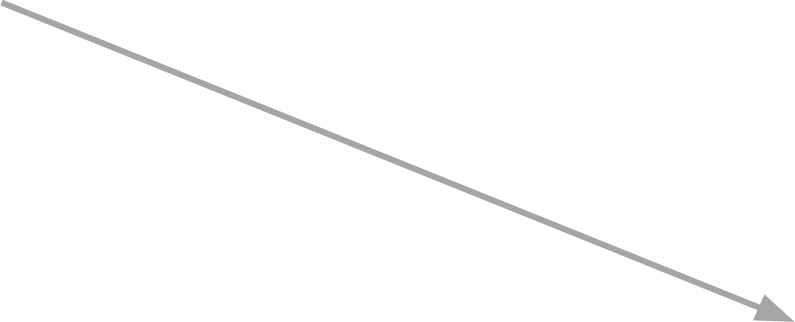

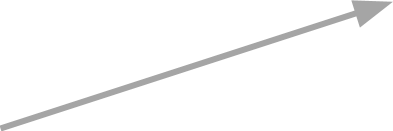

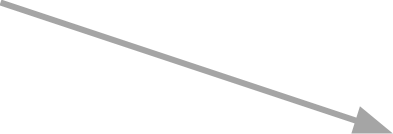


Un-diagnosed

Favourable service outcome (on treatment or completing treatment)

Persons diagnosed


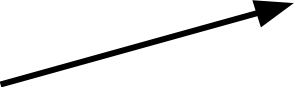

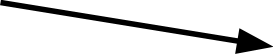

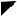

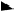


Not favourable service outcome

Persons not diagnosed

Persons not diagnosed

Favourable service outcome (on treatment or completing treatment)


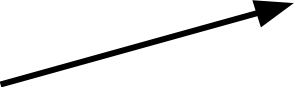

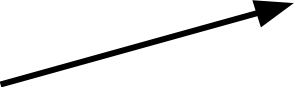


Persons diagnosed

Not favourable service outcome


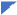

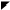

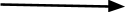
Persons not diagnosed

Persons not diagnosed

Treatment complete


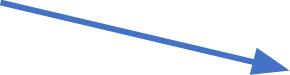
On treatment

Treatment Incomplete

Not on treatment

Not on treatment

Treatment complete


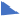
On treatment


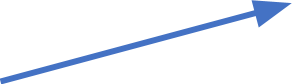

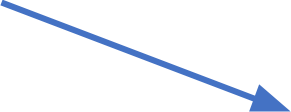


Treatment Incomplete

Not on treatment


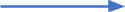

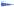

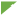

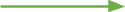
Not on treatment

| Treatment complete | 56 doses |
| --- | --- |


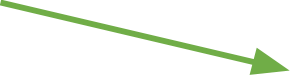
On treatment

Treatment Incomplete

Not on treatment

Not on treatment

Treatment complete


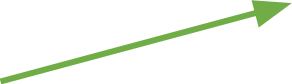

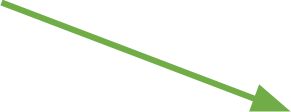


On treatment


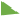
 Treatment Incomplete Not on treatment

Not on treatment

Succssfully on self-care


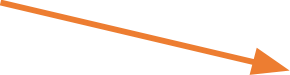
Home based self-care

Progressed to elephantiasis


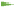

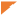

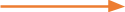

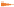
Not on treatment

Not on treatment

Succssfully on self-care


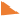
Home based self-care


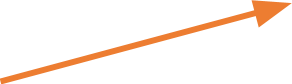

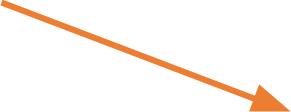


Progressed to elephantiasis

Not on treatment

Not on treatment

Complicated cases with positive outcomes


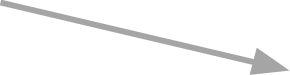
Surgery

Complicated cases with negative outcomes

No surgery


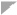

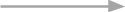

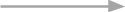
No treatment

Complicated cases with positive outcomes


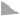
Surgery


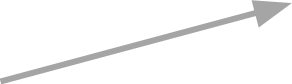

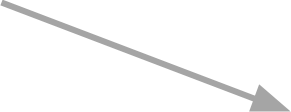


Complicated cases with negative outcomes

Not on treatment

No treatment
