# Supplementary material for: Assessing the cost-effectiveness of integrated case management of Neglected Tropical Diseases in Liberia
Source: BMC Health Serv Res. 2023 Jun 29;23:705. doi: 10.1186/s12913-023-09685-0 (PMC10308665; doi:10.1186/s12913-023-09685-0)
Supplement: Supplementary file 2 — Additional file 2. Analytic assumptions. [file 12913_2023_9685_MOESM2_ESM.docx]

**Analytic assumptions**

The main assumptions of the model are as follows:

- An assumed starting population of 4.8 million, standard population growth of 2.5%

(assuming not all persons survive) for the years 2021-2025 (3).

- Standard prevalence rates for the NTDs of interest are as follows:
  - Assuming 80% of the population is at risk given the country is endemic.
  - For leprosy a prevalence of 24.9 per 10,000 as per (4) o For Buruli ulcer, similarly a prevalence of approx. 2.3% (4) o For lymphatic filariasis assumption of 70% at risk. (13)
- Secondary outcomes were calculated as below; it was assumed that all patients can complete treatment in a given year.
  - Leprosy treatment completion is defined as the total number of leprosy patients (both paucibacillary and multibacillary) recorded to complete treatment in a given year, divided by the number of people diagnosed with leprosy. This assumes an average treatment duration of 6 months to a year and that all diagnoses can be effectively cured within the year. o Buruli ulcer treatment completion is defined as the total number of patients completing a course of 56 antibiotics in a given year, divided by the number diagnosed with the condition in that year. Assuming all patients have the opportunity to complete a course of antibiotics within a given year.
  - Lymphoedema treatment completion is defined as the number of patients on selfcare in a given year, divided by the number of cases newly diagnosed.
  - Hydrocele treatment completion is defined as the number of surgeries for hydrocele conducted in a given year, divided by the total number of confirmed hydrocele patients recorded that year.
- It was assumed the values recorded for costs across Margibi and Grand Cape Mount are likely to be representative of standard costs for MDA and case management across other counties in Liberia.
- It was assumed the cost values recorded across pilot counties be reflective of the total integrated CM-NTDs costs overall, however, we note that laboratory related costs, in particular, are minimally included here and that other costs (e.g. standard salaries of nonincentivized staff who may need to spend time on NTD cases) may also not be captured.
- Overall, we caution that none of the cost estimates include patient incurred costs – either related to hospital stays for hydrocele surgeries or procurement of medications and/or other materials as may need to be bought by patients in case of stock-outs at facility levels.
